# Highly accurate quantification of allelic gene expression for population and disease genetics

**DOI:** 10.1101/gr.276296.121

**Published:** 2022-08

**Authors:** Anna Saukkonen, Helena Kilpinen, Alan Hodgkinson

**Affiliations:** 1 Department of Medical and Molecular Genetics, School of Basic and Medical Biosciences, King's College London, London, SE1 9RT, United Kingdom;; 2 UCL Great Ormond Street Institute of Child Health, University College London, London, WC1N 1EH, United Kingdom;; 3 Wellcome Sanger Institute, Wellcome Genome Campus, Cambridge, Hinxton, CB10 1SA, United Kingdom;; 4 Helsinki Institute of Life Science (HiLIFE), University of Helsinki, Helsinki, 00014, Finland;; 5 Faculty of Biological and Environmental Sciences, University of Helsinki, Helsinki, 00014, Finland

## Abstract

Analysis of allele-specific gene expression (ASE) is a powerful approach for studying gene regulation, particularly when sample sizes are small, such as for rare diseases, or when studying the effects of rare genetic variation. However, detection of ASE events relies on accurate alignment of RNA sequencing reads, where challenges still remain, particularly for reads containing genetic variants or those that align to many different genomic locations. We have developed the Personalised ASE Caller (PAC), a tool that combines multiple steps to improve the quantification of allelic reads, including personalized (i.e., diploid) read alignment with improved allocation of multimapping reads. Using simulated RNA sequencing data, we show that PAC outperforms standard alignment approaches for ASE detection, reducing the number of sites with incorrect biases (>10%) by ∼80% and increasing the number of sites that can be reliably quantified by ∼3%. Applying PAC to real RNA sequencing data from 670 whole-blood samples, we show that genetic regulatory signatures inferred from ASE data more closely match those from population-based methods that are less prone to alignment biases. Finally, we use PAC to characterize cell type–specific ASE events that would be missed by standard alignment approaches, and in doing so identify disease relevant genes that may modulate their effects through the regulation of gene expression. PAC can be applied to the vast quantity of existing RNA sequencing data sets to better understand a wide array of fundamental biological and disease processes.

Allele-specific expression (ASE) is the imbalanced expression of the two alleles of a gene. Whereas many genes are expressed equally from both alleles, gene regulatory differences driven by genetic changes (i.e., regulatory variants) frequently cause the two alleles to be expressed at different levels, resulting in allele-specific expression patterns. With RNA sequencing (RNA-seq) data, the expression from the two alleles can be distinguished and quantified, but this analysis remains susceptible to many technical challenges, despite improved analytical methods (Castel et al. 2015; van de Geijn et al. 2015). To date, ASE analysis has largely been performed in the context of expression quantitative trait loci (eQTL) studies, as an alternative method to identify and characterize effects of regulatory variants on gene expression (Lappalainen et al. 2013; The GTEx Consortium 2020). In these studies, large sample sizes help mitigate the effects of technical biases (Panousis et al. 2014). However, the power of ASE analysis lies in its applicability to individual samples, particularly in the context of rare diseases and other cases where looking at combined haplotypic effects of multiple variants is necessary. Numerous studies have now leveraged the power of ASE in detecting genetic effects on gene regulation (Kumasaka et al. 2016; Findley et al. 2021) and identifying regulatory dysfunction in rare disease samples (Cummings et al. 2017; Mohammadi et al. 2019). In order to draw conclusions about individual samples and loci, the accuracy of ASE calling becomes paramount.

One of the main sources of bias in ASE analysis is the alignment of sequencing reads. When short reads are aligned to the reference genome, reads carrying alleles that match to the reference sequence frequently map better than those carrying alternative alleles, leading to false ASE effects (‘reference allele bias’). This effect can be particularly problematic in areas containing a large number of single nucleotide polymorphisms or insertion/deletion events, which can mean that certain regions of the genome are not fully characterized. Similarly, reads that align to multiple locations in the genome can also influence the accuracy of allele counts at heterozygous sites. In the absence of better approaches, such difficult-to-map reads are typically discarded from the analysis (van de Geijn et al. 2015), and consequently sets of genes that contain similar sequences (e.g., within gene families) may suffer from artificially low sequencing coverage and lack of power to detect the effects of genetic regulation. Diploid genome mapping (i.e., use of personalized genome references) has been proposed as a solution to alignment-related artifacts in ASE analysis (Rozowsky et al. 2011; Dobin et al. 2013), but these approaches do not deal with all potential causes of bias, such as differences in the uniquely mappable genome between parental haplotypes (van de Geijn et al. 2015), and they have not been widely adopted, likely due to the lack of a comprehensive pipeline to handle personalized genome coordinates in downstream analysis. Collectively, these problems can inhibit the detection of genes that are under the influence of perturbed or altered regulatory regimes, which may be particularly limiting when trying to understand the underlying etiology of disease.

Here, we address some of the remaining challenges in ASE analysis and describe the Personalised ASE Caller (PAC) tool that integrates a series of existing and novel methods to improve the detection of genuine ASE events in short read RNA-seq data. PAC deals with the issues outlined above by implementing parental genome mapping to limit the effects of genetic variation on read alignment and reallocating multimapping reads based on uniquely mapped read coverage to limit the removal of informative sequencing reads. PAC also uses improved read-based phasing approaches to ensure that alleles at heterozygous sites are on the correct genetic background, further improving our ability to accurately align data across these regions, and PAC compares alignment scores between parental genomes for each read, minimizing problems that may arise from aligning data to two different genomes. Finally, these steps are applied within an easy-to-use workflow system that produces allele counts at the single site (converted back to reference genome coordinates) and gene level, which can be easily used in downstream analyses.

## Results

**Figure 1.**
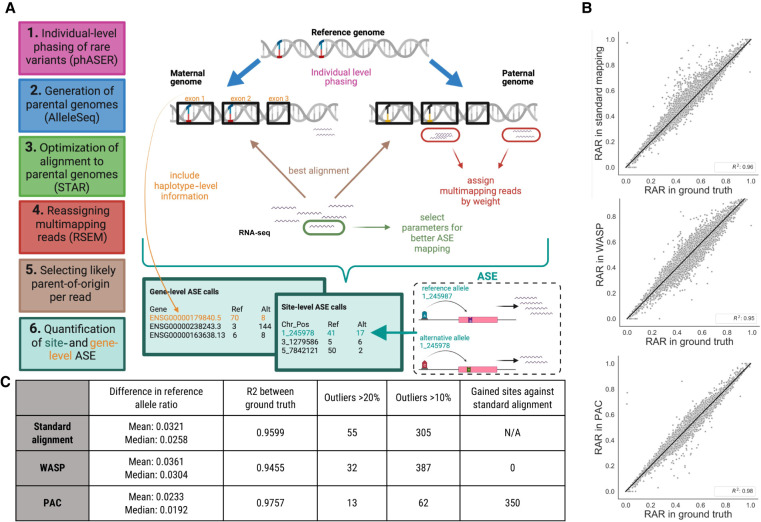
Overview of the PAC pipeline. (*A*) A schematic describing the main steps, features, and outputs of PAC. (*B*) Correlation of reference allele ratios (RARs) between the three different methods (standard alignment, WASP-filtered alignment, PAC) and the ground truth data. Genome-wide Pearson correlation coefficients (R^2^) are shown (*P* < 0.05 for all comparisons). (*C*) Site-level summary statistics for the different analysis methods. Statistics are reported for sites with at least 20× coverage in all three methods. Panel *A* was created with BioRender (https://biorender.com).

In general, PAC implements the following series of steps for each RNA-seq sample to detect and quantify ASE events (Fig. 1A). First, PAC uses phASER (Castel et al. 2016) with read-aware mode to improve variant phasing, particularly for rare variants, before creating parental genomes for the individual with the AlleleSeq software (Rozowsky et al. 2011). RNA-seq reads are aligned to each genome individually with STAR (Dobin et al. 2013), retaining only properly paired and uniquely mapped reads, followed by a realignment step with RSEM (Li and Dewey 2011), which allows for the retention of multimapped reads through the implementation of an expectation-maximization algorithm. For each pair of reads, PAC then selects the best location across the two parental genomes with custom scripts, before site-level and gene-level (phASER) allele counts are extracted genome-wide (see Methods). Throughout the development of PAC, we tested the effects of different read trimming and alignment parameters before arriving at the final optimized pipeline (see Supplemental Materials).

To compare the performance of PAC with other approaches to quantify allelic expression, we generated simulated RNA-seq data where we knew the exact allele counts at each heterozygous site of a single individual (NA12877). To do this, we used high-quality genotype data from the Platinum Genomes Project (Eberle et al. 2017). First, whole-genome sequencing data were simulated using these variants, which were subsequently aligned to the reference genome before variant calling was performed with GATK (Van der Auwera et al. 2013), followed by phasing with SHAPEIT2 (see Methods; Delaneau et al. 2013). In this way, we generated a realistic set of variant calls that could be used for ASE analysis. Second, we generated simulated RNA-seq data for the individual using RSEM. To do this, we simulated RNA-seq data for each parental haplotype inherited by the offspring (based on real RNA-seq data for the parents), before merging and calculating the ‘ground truth’ allele count for each allele at each heterozygous variant position identified in the GATK output (Supplemental Fig. 1). This ground truth data was used as the baseline to which the accuracy of ASE calls obtained using different parameters and methods was compared. In total, the simulated RNA-seq data consisted of ∼69 million read pairs and had a coverage of at least 20× at 13,211 unique heterozygous sites (including 499 rare variants with <1% minor allele frequency in the CEU population from the 1000 Genomes data), 1359 of which (10.3%) showed ASE under a standard binomial test (*P* < 0.05, corrected for 13,211 tests) (Supplemental Fig. 2). Simulated data also contained 1237 indels (>1 bp) with at least 20× coverage.

Simulated RNA-seq reads were then aligned to the reference genome using three approaches: (1) standard alignment using STAR (to obtain a baseline set of ASE calls when using one of the most commonly used haploid alignment methods); (2) PAC (diploid alignment); and (3) WASP (van de Geijn et al. 2015), a commonly used tool to correct for reference bias in ASE analysis. WASP incorporates a number of features to identify sites under ASE, and although we cannot directly compare these with the other approaches, we can test the impact of read removal within the WASP pipeline. After applying each of the three approaches, we counted reads containing the two alleles at each heterozygous site and compared these data to the ground truth (Fig. 1B). We focused on sites that had at least 20× coverage in both the ground truth data and all three alignment approaches (11,602 heterozygous sites) to make results directly comparable (Fig. 1C).

First, with standard alignment, ∼91.7% of sites that had at least 20× coverage in the ground truth data also met this coverage threshold (12,109/13,211), with the average coverage at these sites dropping from ∼175× in ground truth data to ∼144× with standard alignment, highlighting the loss of many reads when using the standard approach. We find that reference allele ratios (RARs) showed a high correlation between standard alignment and the ground truth data at heterozygous sites (R^2^= 0.960) (Fig. 1B). However, 305 sites show an absolute difference in RARs of >10% and 55 sites a difference >20%. The absolute mean difference shows a 3.21% bias across all heterozygous sites (Fig. 1C).

Second, when we applied PAC to the simulated data, we found that the number of reads and the accuracy of allelic assignment was significantly improved compared to standard alignment. First, the number of sites that have at least 20× coverage in both ground truth and PAC-aligned data increased to 12,448 (339 additional sites compared to standard alignment), with an average coverage of ∼150× at these sites. Second, the correlation of RARs in PAC compared to the ground truth data increased to R^2^= 0.976 (Fig. 1B). The number of outlier sites also decreased to 62 and 13 sites, showing an absolute difference in reference allele ratio of >10% and >20%, respectively. The mean difference from ground truth RAR is 2.33% (Fig. 1C), which is significantly lower than that found for standard alignment at the same sites (one-sided *t*-test, *P* = 2.6×10^−125^).

Third, we compared WASP-filtered data to the ground truth and found that the number of sites that have at least 20× coverage in both ground truth and WASP-corrected data dropped to 11,612 (836 fewer than when using PAC), with an average coverage of 135×. Both of these results are likely a consequence of the approach used in WASP to remove difficult-to-align reads from the analysis. Whereas the number of extreme outliers reduced to 32 (absolute difference of >20%) using WASP-filtered data, the number of sites with an absolute difference in RAR of >10% increased to 387 compared to standard alignment, and the R^2^ value decreased to 0.946 (Fig. 1B). Furthermore, the mean absolute difference between WASP-filtered data and the ground truth (3.61%) is significantly higher than both standard alignment (*P* = 4.5 × 10^−21^, one-sided *t*-test) and PAC (*P* = 8.9 × 10^−272^, one-sided *t*-test) (Fig. 1C).

**Figure 2.**
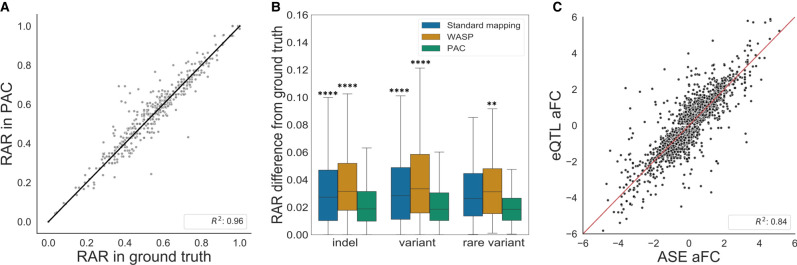
Performance of PAC compared to other methods. (*A*) Genome-wide correlation of reference allele ratios at heterozygous sites that PAC and standard alignment detect but that are discarded by WASP-filtering (Pearson's correlation R^2^ = 0.956, *P* = 2.6 × 10^−266^). Sites with at least 20× coverage were considered. (*B*) The difference in reference allele ratio of sites that are within 500 bp of an at least 6-bp indel, within 25 bp of another variant or a rare (MAF < 1%) variant in different analyses against the ground truth. Sites shared between all methods and with at least 20× coverage were considered. A Mann–Whitney *U* test was performed with Bonferroni correction to adjust for multiple testing. (****) *P* ≤ 1 × 10^−4^, (**) 1.00 × 10^−3^ < *P* ≤1.00 × 10^−2^ , and stars *above* each box plot refer to the comparison against PAC. (*C*) Correlation of allelic fold change (aFC) values derived from ASE and eQTL analyses from 670 GTEx whole-blood samples. Genes with a significant eQTL (*Q*-value < 5%) and gene-level ASE information for at least 10 individuals were selected. Pearson correlation coefficients are shown for eQTL versus ASE aFCs derived using PAC (see also Supplemental Fig. 3).

Compared to both standard and WASP-filtered alignment, applying PAC results in an additional 350 heterozygous sites that have coverage of at least 20× that do not meet this threshold in both of the other two approaches. Allele count quantification at these sites is also highly accurate with PAC (R^2^= 0.844, compared to the ground truth). Similarly, for sites detected in standard alignment and PAC (but not WASP-filtered data), PAC quantification is highly significant (496 sites, R^2^= 0.956, *P* = 2.6 × 10^−266^) (Fig. 2A), showing that PAC performs well at sites with lower coverage that may be missed by other approaches. At least part of the improvement in accuracy when using PAC appears to occur in regions of the genome where accurate alignment is known to be more difficult. For example, the difference in RAR from the ground truth is significantly higher in standard alignment and WASP-filtered data than PAC at sites where there is an indel (>6 bp) within 500 bp of the heterozygous site (Fig. 2B). The trend is similar when another heterozygous site or a rare variant (MAF < 1%) is close by (Fig. 2B).

On the read level, PAC aligns ∼2.4 million read pairs that are not retained by either standard alignment or after WASP-filtering, and 84,045 of these reads align across a heterozygous genetic variant and are therefore informative for ASE. Of these reads, PAC places the read at the exact correct location on the reference genome 86.3% of the time, showing that the vast majority of additional reads aligned by PAC are accurate. Additional reads that are aligned by PAC are not biased towards any particular chromosome and have similar GC content to reads aligned by standard alignment (47.8% vs. 49.1%, respectively), and although 68 genes show a twofold difference in the number of reads aligned between PAC and standard alignment (in either direction), these genes are not enriched for any specific GO functional terms. Finally, because the original simulated data contain a large number of reads, we tested the performance of PAC at lower coverages by randomly resampling simulated raw data to 70% (∼48 million read pairs), 50% (∼34.5 million read pairs), and 30% (∼21 million read pairs) of the original depth, and then compared results to the ground truth in each data set across the three methods. As before, we find that PAC outperforms both standard alignment and WASP-filtered data in terms of the correlation in RAR with the ground truth and the number of outliers at all coverage levels (see Supplemental Table 1 for details).

To characterize the performance of PAC on population level data, we aligned 670 whole-blood RNA-seq samples from the GTEx project (v8) (The GTEx Consortium 2020) using PAC, obtained gene-level counts for each gene and individual, and then compared the allelic fold change (aFC) (Mohammadi et al. 2017) for each gene generated from the count data with those obtained from eQTL mapping. We also compared these results to those generated through standard and WASP-filtered alignments using gene-level count data from Castel et al. (2020). Using genes with a significant eQTL (*Q*-value < 5%) and where the aFC could be calculated from ASE data in all three methods (8913 genes) (see Methods), PAC shows the strongest correlation between ASE and eQTL aFC (R^2^= 0.842) (Fig. 2C), followed by WASP-filtered data (R^2^= 0.829) and then standard alignment (R^2^= 0.820). Furthermore, due to the higher coverage obtained when aligning data with PAC, we were able to generate aFC for an additional 740 genes using this approach that did not meet coverage criteria in WASP-filtered data; the aFC generated from ASE and eQTL data was still highly correlated among these genes (R^2^= 0.653, *P* = 4.0 × 10^−91^), and therefore the additional data generated with PAC is likely to be informative. Similarly, there were 319 genes present in PAC data that were not in either standard or WASP-filtered alignments; again the correlation between ASE and eQTL aFC for these genes was significant (R^2^= 0.643, *P* = 1.1 × 10^−38^). These results indicate that PAC not only has improved accuracy, but due to the higher coverage obtained, PAC also allows analysis of the influence of regulatory variation on gene expression across a larger number of genes.

Finally, to demonstrate the utility of PAC in finding biologically informative events, we attempted to identify cell type–specific ASE using gene expression data from Findley et al. (2021), who exposed three different cell types (lymphoblastoid cell lines [LCLs], induced pluripotent stem cells [iPSCs], and iPSC-derived cardiomyocytes [CMs]) from six individuals to a range of treatments before performing RNA sequencing on each sample. Previous work using these data found that ASE events that were conditional on treatment and/or cell type (cASE) were enriched among genes linked to several disease-relevant human phenotypes. For coronary artery disease (CAD) in particular, where CMs are a highly relevant cell type, metal treatments such as cadmium generated the largest overlap between cASE and putative disease genes (seven genes) (Findley et al. 2021), which is consistent with the role of cadmium in promoting atherosclerosis (Messner et al. 2009). To explore these relationships further, we obtained RNA sequencing data from cells treated with cadmium from six individuals across three cell types, aligned the data with both PAC and standard alignment, and then identified cell type–specific ASE at the site level in CMs. The purpose of this analysis was not to follow up on the original results in Findley et al. (2021) but instead to identify any differences that occur when using PAC versus standard approaches in a biologically interpretable system. Using PAC, we find an average of 102 sites that show a significant bias in allele expression in CMs (binomial test, *P* < 0.05/18,537 tests, which is the mean number of sites tested per sample across all methods and individuals), but not in LCLs or iPSCs (binomial test, *P* > 0.05 uncorrected), and 13 of these sites fall within genes that have been previously linked with CAD (Zhang et al. 2020). Using standard alignment, the average number of CM-specific ASE events per individual is the same as PAC using the same criteria (N = 102); however, many of the sites identified are different, and in total eight sites overlap putative CAD genes. Focusing specifically on sites that show biased allele expression in CMs in PAC data only (as above), we identify four genes (*GPX1*, *RETREG3*, *TCTA*, and *PMVK*) implicated in CAD that would be missed under these criteria using standard alignment approaches.

## Discussion

In summary, we present PAC, a new tool for ASE analysis that generates highly accurate allele counts from RNA-seq data for use in studying the regulation of gene expression. Our approach incorporates both novel and existing analytical steps to better control for common technical biases that occur when aligning short read data, including reference allele bias and reads that align to multiple different genomic locations. Additionally, PAC maximizes ASE quantification accuracy for individual samples by improved phasing of rare variants and subsequent diploid genome alignment.

Using simulated RNA-seq data, we show that PAC performs better than standard alignment techniques and other commonly used tools that attempt to deal with some of the technical issues related to ASE analysis, producing both more accurate allele counts and higher coverage at heterozygous sites. We also show that the additional reads aligned by PAC (and not other approaches) are highly accurate and are not just adding noise to the data. Applying PAC to real population level data (670 whole-blood samples from the GTEx project), we show that genetic regulatory signatures inferred from ASE data closely match those from population-based methods (i.e., eQTL mapping) that are less prone to alignment biases. Further, PAC increases the aligned sequencing coverage compared to standard alignment or WASP-filtered data, allowing for the quantification of allelic fold change for a larger number of genes that also show good correlation with population level signatures, showing that PAC improves the detection of genuine signals. Finally, we use PAC to identify gene-by-environment interaction effects in CMs treated with cadmium and show that some genes identified using PAC (but missed by other methods) overlap with genes linked to cardiovascular disease. These results could be important in understanding the roles of these genes in the underlying etiology of the disease, where the impact of regulatory variation on gene expression may affect disease risk.

Although PAC improves the accuracy of allelic quantification, its use comes with several limitations. First, due to its diploid mapping approach, PAC requires longer processing times than standard alignment methods. Using five test GTEx samples of average depth of ∼44 million paired reads, PAC takes an average of 12 h and 6 min to generate site- and gene-level ASE data per sample, whereas generating these data from standard alignment takes an average of 3 h and 28 min on the same computational set up. Although this is a longer time, we do not believe that it is prohibitive for a reasonable number of samples. Second, PAC requires phased genetic variants to construct parental genomes for the alignment of data. This may be obtained from whole genome/exome sequencing, or from a genotyping array of the same sample, but it is not inconceivable that using genetic variant calls generated from the same RNA sequencing sample would also lead to improved allelic quantification within PAC. Third, the utility of PAC to identify biologically meaningful signatures will, of course, depend on the sample and question under study and therefore will not always lead to greater insight when compared to data generated from standard approaches. However, here, we show that PAC generates improved allelic quantifications and results when tested against two different reference genomes (GRCh37 and GRCh38), at multiple different coverage thresholds (across RNA sequencing data from simulated data, population level experiments, and treated cell types) and in different biological settings, showing the potential widespread utility of the tool. Fourth, PAC is only able to correct alignment errors in RNA sequencing data and has no effect on technical biases introduced at other stages of data generation, including those that may have arisen during library preparation (such as amplification biases).

We anticipate PAC to be useful primarily in the context of rare diseases and other situations where small sample size precludes the use of population level methods to study differences in gene expression and regulation. In such cases, accurate quantification of allelic expression changes, in individual samples, is of paramount importance in understanding disease biology. However, PAC can also be used on population level data, where allelic imbalance information can be used to better infer the impact of genetic variants on the expression of nearby genes. In these ways, PAC can be applied to the vast quantity of existing RNA-seq data sets to better understand a wide array of fundamental biological and disease processes.

## Methods

### Ground truth variant calls

Ground truth variant data was obtained for a single individual from the Platinum Genome Project (PGP), specifically, NA12877 from CEPH/Utah pedigree 1463 (Eberle et al. 2017). The PGP generated deep (50× average) whole-genome sequencing (WGS) data from 17 individuals in a three-generation pedigree, using two different sequencing technologies and variant calls from six different informatics pipelines. Conflicts between call sets were resolved using inheritance-based validation. This data set is widely considered to represent the most accurate set of variant calls that can be achieved with current methods. We used phased variant calls (VCF file) for NA12877 that included indels and SNPs to generate a diploid genome reference using AlleleSeq (Rozowsky et al. 2011) and the hg19 version of the human reference genome.

### Simulated WGS data

The maternal and paternal genomes from AlleleSeq were used to simulate whole-genome sequencing reads for NA12877 with ART (Huang et al. 2012). As input, ART requires parameters related to insert size, read length, coverage, and standard deviation of fragment length. To obtain a realistic simulation, we acquired these parameters from real sequencing data for sample HPSI0114i-eipl_1 from the HipSci project (Kilpinen et al. 2017). WGS reads for ‘eipl_1’ were aligned to the hg19 reference genome using BWA-MEM (Li and Durbin 2009). SAMtools (Li et al. 2009) was then used to limit the output to uniquely mapping, properly paired reads and to obtain the required parameters for ART (from *samtools-stats*). Parameters were set as follows: (1) 20× coverage; (2) read length 150 bp; (3) mean fragment length 479 bp; and (4) standard deviation of fragment size 117 bp. The maternal and paternal reads were simulated separately and then merged so that the final coverage of the simulated WGS sample was 40×.

### Variant calling

Simulated WGS reads were aligned to the reference genome (hg19) the same way as described above, and variant calling was performed with GATK v. 4.0.12.0 according to recommended best practices (Van der Auwera et al. 2013). Variants were then phased with SHAPEIT2 (Delaneau et al. 2013) using the 1000 Genomes phase 3 reference panel. These variants were then compared against the ground truth variants from PGP (see Supplemental Results).

### Simulation of RNA sequencing data

In order to construct ground truth allele counts for NA12877, we simulated RNA sequencing reads using RSEM v1.3.1 (Li and Dewey 2011). To obtain realistic input parameters for RSEM, we used real data from the parents of this individual (NA12889 and NA12890) (Supplemental Fig. 1). Raw RNA-seq data for the parents (from lymphoblastoid cell lines) were obtained from the Geuvadis Project (Lappalainen et al. 2013), trimmed, and mapped to the hg19 reference genome using STAR v.2.5.1a (Dobin et al. 2013) with default parameters. The mapped reads from the parents (two separate BAM files) were then input into RSEM to generate a single matrix of expression levels for each transcript in the GENCODE v19 annotations. To simulate RNA-seq reads, the *rsem-simulate-reads* function was used with the following input parameters obtained from the alignment of data from NA12889 and NA12890: (1) fraction of reads coming from background noise (0.27 and 0.19, paternal and maternal sample, respectively); and (2) total number of reads to be simulated (40.9 M and 28.1 M paternal and maternal sample, respectively). Because the input for the simulations is based on real data from two distinct individuals, this generates allelic variation at heterozygous positions of the genome, that is, ASE effects in the simulated data. To acquire ‘ground truth allele counts,’ we obtained maternal and paternal allele counts at heterozygous genome positions of NA12877 from the PGP VCF file. The simulated reads from the parents (FASTA files) were then merged into a single RNA-seq sample, representing the (simulated) transcriptome of individual NA12877.

### Ground truth allele counts

The genomic coordinates of the simulated RNA-seq reads were obtained using custom scripts, based on the flag information stored for each read by RSEM (including transcript ID, position on transcript, etc.). liftOver was used to convert read locations from the parental genomes to the reference genome using chain files generated by AlleleSeq. We then counted the number of reads from reference and alternative alleles that overlapped all heterozygous positions in NA12877 based on the PGP variant calls. These allele counts were then combined for each site to create ground truth allele counts in the offspring. For all subsequent analyses, we used heterozygous sites with at least 20× read coverage (sum of reference and alternative allele counts). The distribution of the reference allele ratios across all 20× sites in the ground truth data is shown in Supplemental Figure 2.

### Standard alignment of simulated RNA-seq reads

The paired-end reads were aligned to a reference genome (1000G version of GRCh37) with STAR 2.51a with standard parameters including soft-clipping, using two-pass mapping, version 19 of the GENCODE gene annotation, and allowing eight mismatches per read pair, before keeping only properly paired (-f 0 × 0002 using SAMtools) and uniquely mapped (NH:i:1 flag) reads.

### PAC pipeline

The final PAC pipeline (Fig. 1) was constructed as follows, with each step tested for its impact on the accurate alignment of reads compared to the ground truth data (see Supplemental Table 2). First, standard alignment of RNA-seq reads was performed (see above). These data were then used as input for phASER (Castel et al. 2016), alongside the phased VCF obtained from the GATK pipeline above, to redetermine the phase at heterozygous sites where RNA-seq reads can add information (read-aware mode). The resulting VCF file was then used with AlleleSeq (Rozowsky et al. 2011) to generate parental genomes. For each parental genome, simulated RNA-seq reads were aligned with STAR as above, keeping only properly paired and uniquely aligned reads. Because the uniquely alignable regions in the reference and nonreference genome may differ, we also used RSEM (v1.3.0) (Li and Dewey 2011) to take the original alignment from STAR (containing all reads aligned to transcriptome coordinates, including reads that align to multiple locations) and realigned the data using the ‐‐sampling-for-bam flag to output a single location for each read based on its posterior probability generated from estimated abundances. Additional reads aligned by RSEM that were not uniquely aligned using STAR were then added to the final BAM file. After alignment of each parental genome, a custom script was used to select the best alignment for each read from the two mappings (scoring reads by the number of matching nucleotides minus two times the number of indel positions, drawing at random when the two mappings have equal scores), and the number of each allele at each heterozygous site was counted. We also produce allele counts at haplotypic level using phASER Gene AE.

### WASP

WASP-filtering was performed using the same approach as detailed above for standard alignment but using the additional flag ‐‐waspOutputMode SAMtag within STAR (v2.7.3a), together with providing the VCF file generated from GATK (as described above). We filtered the resulting BAM file for reads that were properly paired, reads without a WASP flag (and thus do not contain a genetic variant), and reads that pass WASP-filtering (with flag ‘vW:i:1’), before counting reference and alternative alleles at heterozygous sites.

### Evaluation of allele count accuracy/outlier analysis

In order to evaluate the performance of the pipeline and how the different steps influence the accuracy of allele counts (and eventual ASE calls), we compared results obtained with PAC, standard alignment, and WASP-filtering to the ground truth data. We excluded sites that were located in the HLA region as well as blacklisted genomic regions (obtained from phASER). We monitored the number of ‘accessible’ heterozygous sites (i.e., biallelic sites that had at least 20× coverage, obtained through SAMtools *mpileup* using default parameters and disabling read-pair overlap detection), correlation of the reference allele ratios in the analysis and the ground truth, sites that were present in standard alignment but missed by analysis, and the number of sites where the RAR showed more than 10% or 20% difference between the analysis and the ground truth (referred to as outliers).

### Accuracy of analysis near indels and other variants

To evaluate how PAC performs at genomic regions that are difficult to align relative to standard alignment and WASP-filtered data, we compared the difference in RAR against ground truth RARs at heterozygous sites. For indel analysis, we selected sites that were within 500 bp of an indel (minimum indel length 6 bp). We also looked at sites that had another heterozygous single nucleotide variant or rare variant (MAF < 1%) within 25 bp of the heterozygous site. We used CEU population data from the 1000 Genomes Project for this. A Mann–Whitney *U* test was performed with Bonferroni correction to adjust for multiple testing (Fig. 2B).

### Analysis of GTEx samples

To interrogate the performance of PAC on population level data, we obtained aligned data containing all reads for 670 whole-blood samples from the GTEx project (v8, aligned to the hg38 reference genome, obtained from the GTEx Portal and the NCBI database of Genotypes and Phenotypes [dbGaP; https://www.ncbi.nlm.nih.gov/gap/] accession number phs000424.v8.p2) (The GTEx Consortium 2020), converted these files back to raw sequence files (FASTQ) with SAMtools, and then used them as input for PAC (selecting the GRCh38 reference genome), together with phased genetic variant calls from WGS data (obtained from the GTEx, phASER_GTEx_v8_merged.vcf.gz). GTEx samples have an average of 9972 (SD = 3809) heterozygous variants covered by at least 20 reads, with at least one read present for each nucleotide. Gene-level count data was then obtained from the output of PAC and used to calculate allelic fold change estimates per gene using phASER-POP (Castel et al. 2020), which retains only genes and samples with at least eight read counts. Within phASER-POP, we supplied lead eQTL variants identified in the GTEx project (v8) for each gene, which we obtained from the GTEx portal (Whole_ Blood.v8.egenes.txt.gz). We also ran phASER-POP using two additional gene count matrix files representing standard alignment and WASP-filtered alignment, both obtained through the GTEx portal (phASER_GTEx_v8_matrix.gw_phased.txt.gz and phASER_WASP_GTEx_v8_matrix.gw_phased.txt.gz, respectively) and produced by Castel et al. (2020). We then compared the aFC for each gene generated using ASE data where at least 10 individuals were heterozygous for the lead eQTL variant linked to the gene, with aFC estimates generated from eQTL data after filtering genes where the eQTL association was *Q*-value < 5%. We selected only genes that were present in all three methods for direct comparison.

### Cell type–specific ASE

Raw RNA sequencing reads generated by Findley et al. (2021) were downloaded for cadmium-treated lymphoblastoid cell lines, induced pluripotent stem cells, and iPSC-derived cardiomyocytes from six individuals from the NCBI BioProject database (BioProject; https://www.ncbi.nlm.nih.gov/bioproject/) under PRJNA694697. Each sample was then used as input for PAC (selecting the GRCh38 reference genome), together with phased genetic variant calls generated from whole-genome sequencing data within the 1000 Genomes Project (The 1000 Genomes Project Consortium 2015), generating site-level allelic quantifications. Standard alignment files were also generated with PAC, mapping with STAR to the same standard reference sequence, and filtered for properly paired and uniquely mapped reads as described above, before site-level allele counts were generated. For each individual and within each method (PAC and standard alignment), we focused on sites with at least 20× coverage in all three cell types and then identified sites that showed significant biases in allele expression in CMs using a binomial test (*P* < 0.05/18,537 tests, which is the mean number of sites tested per sample across all methods and individuals and ensures significance thresholds are comparable across methods) but did not show such biases in either LCLs or iPSCs (*P* > 0.05 in these cases). For sites showing significant ASE in each case, we then compared genes containing the site to those identified as potentially playing a role in coronary artery disease by Zhang et al. (2020).

## Data access

The PAC pipeline, nextflow, Dockerfile, custom scripts, and simulated expression data generated in this study are available at GitHub (https://github.com/anna-saukkonen/PAC) and as Supplemental Code.

## Supplementary Material

Supplemental Material
