# Supplementary material for: Highly accurate quantification of allelic gene expression for population and disease genetics
Source: Genome Res. 2022 Aug;32(8):1565–72. doi: 10.1101/gr.276296.121 (PMC9435737; doi:10.1101/gr.276296.121)
Supplement: Supplemental Material [file supp_32_8_1565__DC1.html]

Highly accurate quantification of allelic gene expression for population and disease genetics — Highly accurate quantification of allelic gene expression for population and disease genetics — Supplemental Material 

# Highly accurate quantification of allelic gene expression for population and disease genetics

## Supplemental Material

- Supplementary\_Code.zip
- Supplemental\_Material.pdf
