# Supplementary material for: Highly accurate quantification of allelic gene expression for population and disease genetics
Source: Genome Res. 2022 Aug;32(8):1565–72. doi: 10.1101/gr.276296.121 (PMC9435737; doi:10.1101/gr.276296.121)
Supplement: Supplemental Material [file supp_gr.276296.121_Supplemental_Material.pdf]

Supplemental Material for "Highly accurate quantification of allelic gene expression for population and disease genetics"

This document contains the following supplementary material for the manuscript:

- Supplementary Results
- Supplementary Figures 1-3
- Supplementary Table 1

Supplementary Results

Assessment of variant calling accuracy on ASE detection

Accuracy of variant calls is a known source of error in ASE analysis. For example, variants erroneously called as heterozygous would show complete monoallelic expression in ASE analysis. Further, while ASE analysis is typically carried out only with SNVs, the call accuracy of short indels and copy number variants (CNV) can influence the mapping of reads when personalised genomes are used. As such, we analysed the ability of GATK (Van der Auwera et al. 2013) to identify heterozygous variants using simulated whole genome sequencing data as described in the methods. In total there are 4,042,773 data points in the PGP VCF file for NA12877 and 4,011,226 in the GATK output, showing a true positive rate of 99.22%. The GATK VCF file also contains an additional 5,389 data records not present in the original data, leading to a false positive rate of 0.134%. The GATK VCF misses 36,939 data points, with false negative rate being 0.914%.

Following this, we generated two parental genomes within AlleleSeq (Rozowsky et al. 2011) using phased DNA variant calls from PGP (Eberle et al. 2017) for NA12877 and aligned simulated RNA-seq data to each genome individually before selecting the best alignment for each read pair for the two mappings (see **Methods**). In the ground truth data there are 13,211 heterozygous sites that have at least 20× coverage. Using this alignment approach, the number of heterozygous sites that also have at least 20× coverage was 12,405, with an average coverage of ~149×, and the correlation between the reference allele ratio at these sites for the aligned data versus the ground truth was R^2=0.962. In total, 161 sites showed an absolute difference in reference allele ratio (versus the ground truth) of greater than 10%, and 72 sites showed a ratio >20% (**Supplementary Table 1**).

Effects of read trimming and softclipping

Many RNA-seq processing pipelines perform read trimming for adaptors, base quality and polyA tails before alignment. In ASE analysis, this may lead to biased results if alternative alleles at the ends of reads are preferentially trimmed. As such, we compared the effects of read trimming against using soft-clipping within STAR (Dobin et al. 2013) on allele counts at heterozygous sites, and found a much closer agreement with the ground truth for the latter approach. Including read trimming (stringency of 3bp, removing adaptors and terminal bases with Phred qualities lower than 30), the number of heterozygous sites with at least 20× coverage in both aligned and ground truth data was 12,161 and the correlation of reference allele ratios versus the ground truth decreased to R^2=0.947. Similarly, the number of sites showing an absolute difference in reference allele ratio of >10% and >20% was 252 and 69, respectively (**Supplementary Table 1**), showing that applying softclipping with STAR is favourable to read trimming in these circumstances.

Effects of local phasing of variants (phASER)

Accurate phasing of genetic variants is required to construct correct parental genomes. However, it is difficult to accurately phase rare alleles if they are not present in reference datasets. As such, we used the read-aware mode of phASER (Castel et al. 2016) within our pipeline, which improves local phasing by considering whether nearby genetic variants fall on the same or opposite reads (or pairs). Using this approach, we see a marginal improvement in all parameters: 12,415 heterozygous sites have coverage of at least 20× in aligned data, the correlation between reference allele ratios increases slightly to R^2=0.963 (versus the ground truth), and the number of sites showing an absolute difference in reference allele ratio between ground truth and aligned data decreases slightly to 157 for differences >10% and 68 for differences >20% (**Supplementary Table 1**).

Effects of recovering multi-mapping reads

In many RNA-seq experiments, reads that do not align uniquely to the reference genome are typically discarded. For ASE analysis, this can lead to biased allele counts at specific loci, if the expression level of one or both alleles is underestimated due to non-unique alignment. Methods to recover reads that align to multiple locations (from hereon 'multi-mapping' reads) exist, but current ASE detection methods do not incorporate these reads into the analysis. In order to include such reads, we used RSEM (Li and Dewey 2011) within our pipeline to assign a single location for multi-mapping reads based on read depth of uniquely aligned reads (see **Methods**) for each parental genome, and then incorporated these reads into the final aligned files before selecting the best alignment for each read pair for the two mappings. Applying this

approach, we again improve the accuracy of the alignment, with 12,448 heterozygous sites having at least 20× coverage, the correlation of reference allele proportion at the sites between the ground truth and aligned data of R^2=0.968, and 140 and 46 sites showing an absolute difference in reference allele ratio of >10% and >20%, respectively (**Supplementary Table 1**).

Supplementary Figures

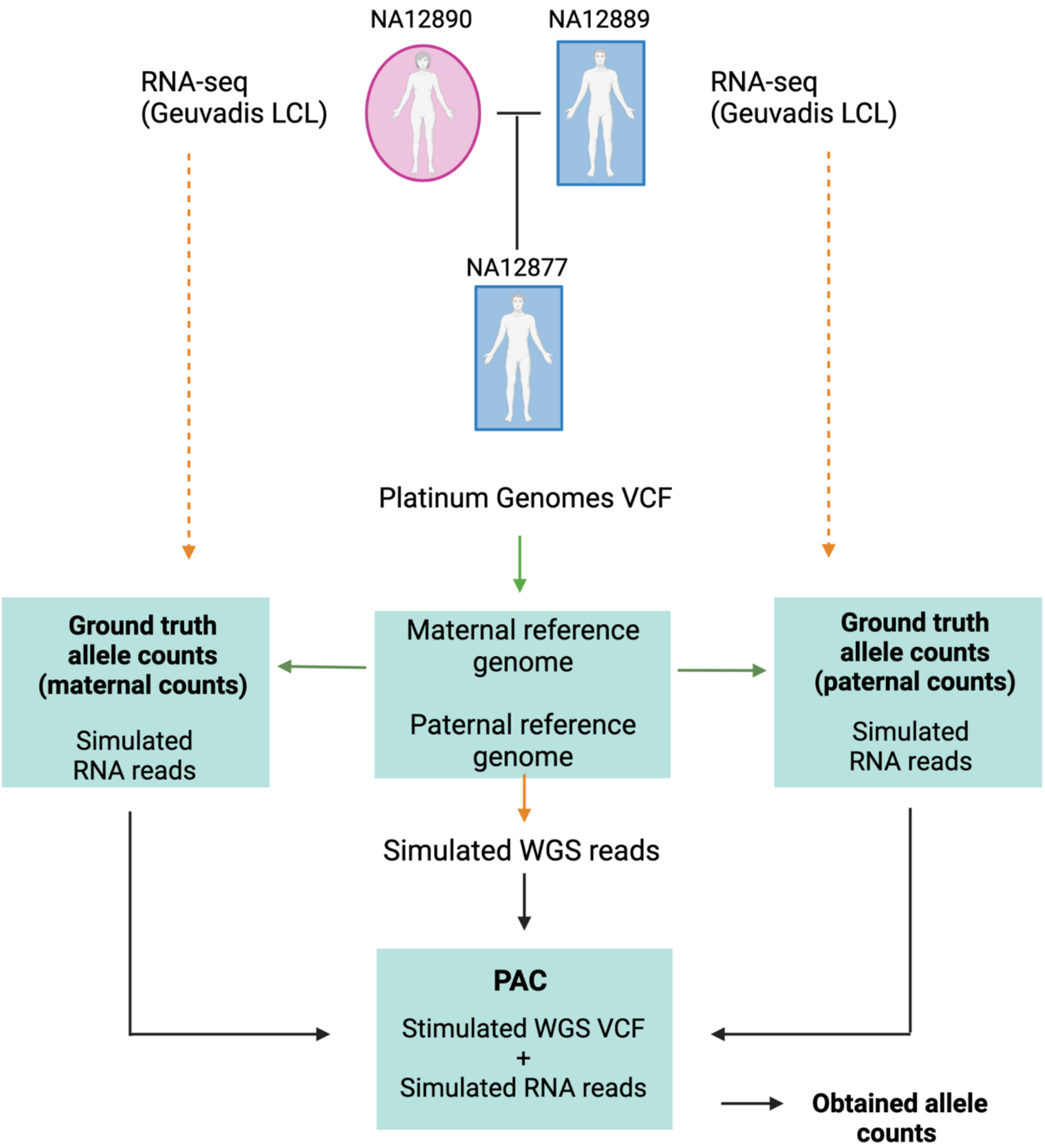

Supplementary Figure 1. Ground truth data generation for individual NA12877. In Platinum Genomes VCF, variants were verified using multiple sequencing platforms and analysis methods, and conflicting calls resolved using parental genomic information. Platinum Genomes VCF together with a reference genome were used to generate ground truth genomes using AlleleSeq. Ground truth genomes were used to simulate RNA sequencing reads using RSEM. Parameters were obtained from RNA-sequencing reads of LCLs from individuals NA12890 and 12889 that were the actual parents, obtained from the Geuvadis Project. The simulated RNA reads were used to count coverage at each heterozygous site, called ground truth allele counts. Ground truth genomes were also used to simulate WGS using ART. Parameters for this were obtained from HipSci sample HPSI0114i-eipl_1. Simulated WGS were used to obtain variant calls using GATK best practises. This VCF, together with simulated RNA-seq reads, were used for PAC to obtain allelic count data that were compared against ground truth allele counts at heterozygous sites.

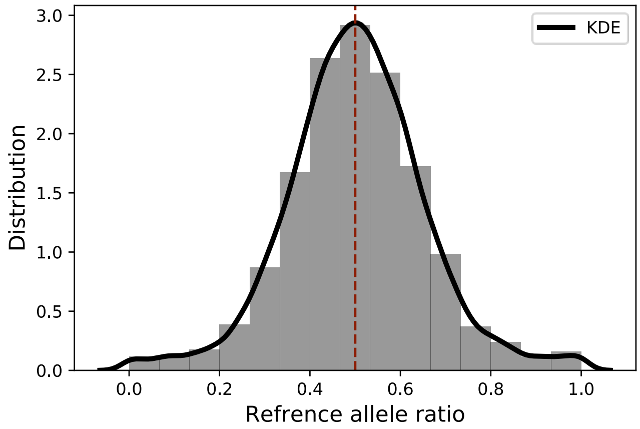

Supplementary Figure 2. Distribution of reference allele ratios in ground truth data. All heterozygous sites in ground truth data with 20× coverage are shown. Ratio of 0.5 implies that both alleles are expressed at equal ratios. KDE = kernel density estimate.

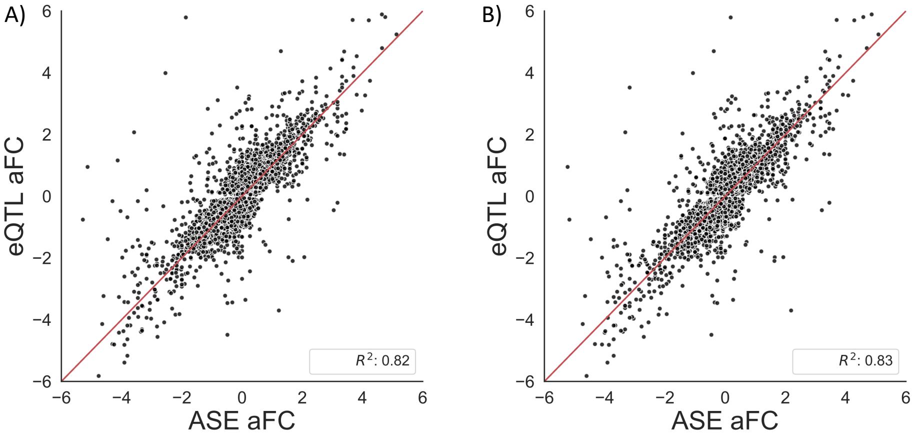

Supplementary Figure 3. Gene-wise comparison of ASE and eQTL signals. Correlation of allelic fold change (aFC) values derived from ASE and eQTL analyses from 670 GTEx whole blood samples. Genes with a significant eQTL (q-value < 5%) and gene-level ASE information for at least 10 individuals were selected. Pearson's correlation coefficients are shown for eQTL versus ASE aFCs derived using standard alignment **(A)** and WASP **(B)**.

Supplementary Table 1. The effects of down sampling simulated data on the accuracy of PAC. Correlation of reference allele ratios (RAR) between the three different methods (standard alignment, WASP-filtered alignment, PAC) and the ground truth data after down sampling RNA sequencing reads to 70%, 50% and 30% of the initial number. Genome-wide Pearson's correlation coefficients (R^2) are shown ($P<0.05$ for all comparisons).

Proportion of sample	1			0.7			0.5			0.3		
Method	Standard mapping	PAC	WASP	Standard mapping	PAC	WASP	Standard mapping	PAC	WASP	Standard mapping	PAC	WASP
Sites shared with ground truth	12109	12448	11612	9984	10356	9271	8124	8452	7411	5454	5791	4888
Difference in reference allele ratio	Mean: 0.0321<br>Median: 0.0258	0.0233<br>0.0192	0.0361<br>0.0304	0.0359<br>0.0296	0.0246<br>0.0207	0.0412<br>0.0343	0.0367<br>0.0306	0.0252<br>0.021	0.0416<br>0.0352	0.0367<br>0.0299	0.0255<br>0.0212	0.0425<br>0.0357
R2 between ground truth	0.96	0.9757	0.9455	0.9541	0.9732	0.93	0.9499	0.9707	0.9244	0.9448	0.968	0.915
Outliers >20%	55	13	32	43	11	24	35	10	18	18	5	12
Outliers >10%	305	62	387	307	61	470	243	56	401	148	28	277
Sites not in standard alignment	-	350	0	-	377	0	-	333	0	-	340	0
Sites not in WASP	497	846	0	713	1089	0	713	1044	0	566	904	0

Supplementary Table 2. Summary of PAC parameter optimisation. Multiple different steps were tested for their impact of allelic read counts, including trimming of adaptors and low quality nucleotides (TRIM), soft-clipping in STAR (SOFT), read-aware phasing in phASER (PHASE) and reallocation of multi-mapped reads (MULTIMAP) (see **methods**).

	TRIM PHASE NO MULTIMAP	TRIM PHASE MULTIMAP	SOFT PHASE MULTIMAP	SOFT PHASE NO MULTIMAP	TRIM NO PHASE NO MULTIMAP	SOFT NO PHASE MULTIMAP	SOFT NO PHASE MULTIMAP	SOFT NO PHASE NO MULTIMAP
Sites shared with ground truth	12159	12194	12448	12415	12161	12190	12436	12405
Difference in reference allele ratio	Mean: 0.0331<br>Median: 0.0273	Mean: 0.0326<br>Median: 0.0273	Mean: 0.0248<br>Median: 0.0195	Mean: 0.0254<br>Median: 0.0196	Mean: 0.0332<br>Median: 0.0273	Mean: 0.0326<br>Median: 0.0272	Mean: 0.0249<br>Median: 0.0196	Mean: 0.0255<br>Median: 0.0196
R2 between ground truth	0.9475	0.9532	0.9681	0.96251	0.9469	0.9530	0.9679	0.9619
Outliers >20%	67	47	46	68	69	49	49	72
Outliers >10%	246	240	140	157	252	244	144	161
Sites not in standard alignment	209	242	350	318	207	233	339	311
Sites not in WASP	606	640	846	813	605	632	833	802

References

Castel SE, Mohammadi P, Chung WK, Shen Y, Lappalainen T. 2016. Rare variant phasing and haplotypic expression from RNA sequencing with phASER. *Nat Commun* **7**: 12817.

Dobin A, Davis CA, Schlesinger F, Drenkow J, Zaleski C, Jha S, Batut P, Chaisson M, Gingeras TR. 2013. STAR: ultrafast universal RNA-seq aligner. *Bioinformatics* **29**: 15-21.

Eberle MA, Fritzilas E, Krusche P, Kallberg M, Moore BL, Bekritsky MA, Iqbal Z, Chuang HY, Humphray SJ, Halpern AL et al. 2017. A reference data set of 5.4 million phased human variants validated by genetic inheritance from sequencing a three-generation 17-member pedigree. *Genome Res* **27**: 157-164.

Li B, Dewey CN. 2011. RSEM: accurate transcript quantification from RNA-Seq data with or without a reference genome. *BMC Bioinformatics* **12**: 323.

Rozowsky J, Abyzov A, Wang J, Alves P, Raha D, Harmanci A, Leng J, Bjornson R, Kong Y, Kitabayashi N et al. 2011. AlleleSeq: analysis of allele-specific expression and binding in a network framework. *Mol Syst Biol* **7**: 522.

Van der Auwera GA, Carneiro MO, Hartl C, Poplin R, Del Angel G, Levy-Moonshine A, Jordan T, Shakir K, Roazen D, Thibault J et al. 2013. From FastQ data to high confidence variant calls: the Genome Analysis Toolkit best practices pipeline. *Curr Protoc Bioinformatics* **43**: 11 10 11-11 10 33.
